# Pathophysiological subtypes of mild cognitive impairment due to Alzheimer’s disease identified by CSF proteomics

**DOI:** 10.1186/s40035-024-00412-1

**Published:** 2024-04-09

**Authors:** Daniela Moutinho, Vera M. Mendes, Alessandro Caula, Sara C. Madeira, Inês Baldeiras, Manuela Guerreiro, Sandra Cardoso, Johan Gobom, Henrik Zetterberg, Isabel Santana, Alexandre De Mendonça, Helena Aidos, Bruno Manadas

**Affiliations:** 1 Faculty of Medicine, University of Lisbon, 1649-028 Lisbon, Portugal; 2 CNC - Center for Neuroscience and Cell Biology, University of Coimbra, 3004-504 Coimbra, Portugal; 3 CIBB - Centre for Innovative Biomedicine and Biotechnology, University of Coimbra, Coimbra, Portugal; 4 LASIGE, Faculty of Sciences, University of Lisbon, 1649-028 Lisbon, Portugal; 5 Biocomputing Group, Department of Pharmacy and Biotechnology, University of Bologna, Bologna, Italy; 6 Faculty of Medicine, University of Coimbra, Coimbra, Portugal; 7 Department of Psychiatry and Neurochemistry, Institute of Neuroscience and Physiology, The Sahlgrenska Academy at the University of Gothenburg, S-431 80 Mölndal, Sweden; 8 Clinical Neurochemistry Laboratory, Sahlgrenska University Hospital, S-431 80 Mölndal, Sweden; 9 Department of Neurodegenerative Disease, UCL Institute of Neurology, Queen Square, London, WC1N 3BG UK; 10 Kong Center for Neurodegenerative Diseases, Clear Water Bay, Hong Kong, China; 11 Wisconsin Alzheimer’s Disease Research Center, School of Medicine and Public Health, University of Wisconsin, University of Wisconsin-Madison, Madison, WI 53792 USA; 12 Department of Neurology, Hospital and University Centre of Coimbra, Coimbra, Portugal; 13 UK Dementia Research Institute at UCL, London, WC1N 3BG UK

The number of patients with Alzheimer's disease (AD) is increasing worldwide due to extended life expectancy, with AD being the most common cause of dementia. AD pathological hallmarks consist of brain depositions of aggregated amyloid beta (Aβ) into neuritic plaques and neurofibrillary tangles of hyperphosphorylated tau, leading to synaptic dysfunction and neuronal loss [1]. Proteomic studies of cerebrospinal fluid (CSF) have shown that several biological processes are dysregulated in AD, such as the innate immune system, inflammatory response, hemostasis, lipid processing, oxidative stress response and synaptic functioning [2]. Some of these alterations may already be present at the early stages of the disorder. Remarkably, a recent study identified three biological AD subtypes based on the CSF proteome of two independent AD cohorts as having hyperplasticity, innate immune activation and blood–brain barrier dysfunction profiles, respectively [3]. Proteomic studies have usually compared AD patients with healthy control subjects; however, patients with AD, even at initial stages corresponding to mild cognitive impairment (MCI), show modifications in lifestyle, changes in diet, weight loss, and presence of comorbidities and drug treatments. As a consequence, metabolic, inflammatory and immune changes might occur that could potentially translate into an altered proteome. The existence of different AD subtypes through CSF proteomics, coupled with a deep understanding of the underlying pathological mechanisms in early stages, holds significant implications for comprehending the disease. It also has profound consequences for the development of disease-modifying treatments, which may need to be tailored to benefit specific subtypes of the disease, eventually being ineffective or even detrimental in others.

The present work (Additional file 1: Fig. S1) represents original features in relation to previous studies, since we (1) focused on the initial phases of AD, that is, patients with MCI within the Cognitive Complaints Cohort (CCC) [4]; (2) recruited patients with MCI who exhibited amyloid and neuronal injury biomarkers indicative of a high likelihood of AD (MCI_AD_; *n* = 45; adapted from the National Institute on Aging—Alzheimer’s Association workgroups [5]); (3) selected a control group of MCI patients without any biomarkers of Aβ deposition or neuronal injury (MCI_Other_; *n* = 23), in order to control for nonspecific changes that might influence the CSF proteome in patients with MCI; and (4) applied the same methodology to MCI patients with (*n* = 92) and without (*n* = 102) AD pathology from the European Medical Information Framework for Alzheimer’s Disease (EMIF-AD) cohort for further validation (Fig. 1a and Additional file 2: Tables S1).

**Fig. 1:**
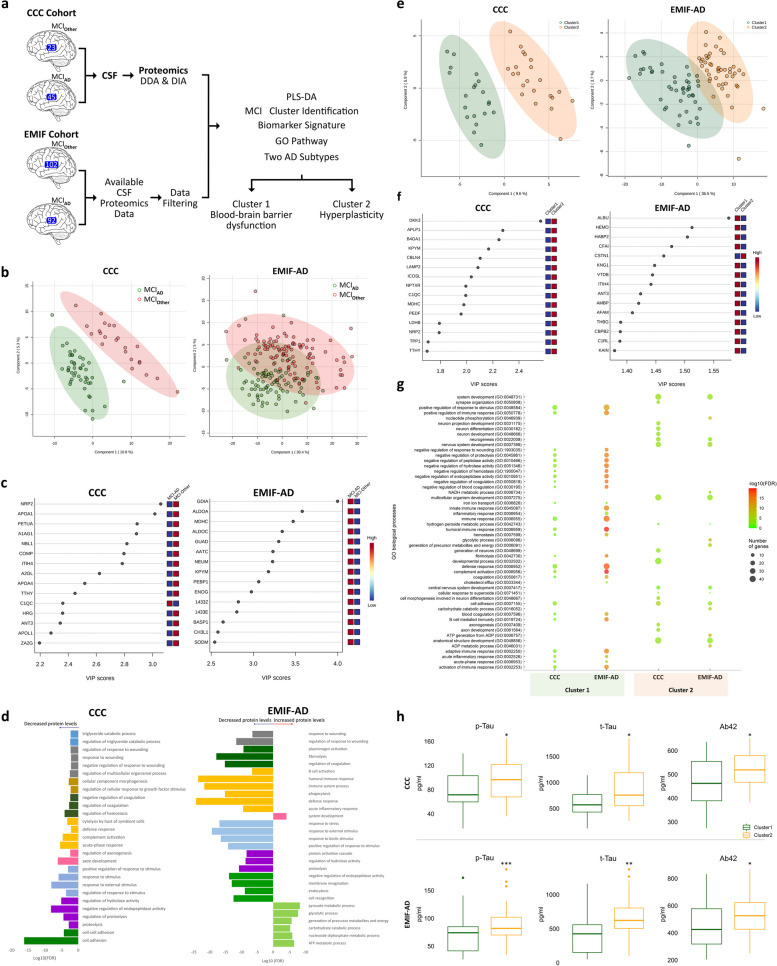
CSF proteomics identifies pathophysiological subtypes of MCI_AD_. **a** Study workflow with CSF samples from 45 MCI_AD_ and 23 MCI_Other_ patients from the CCC cohort. Proteomic characterization and data analysis consisted of PLS-DA followed by MCI_AD_ patient clustering analysis for subsequent subtype characterization through system biology analysis. Two clusters were identified and validated by submitting the proteomic data of 92 MCI_AD_ and 102 MCI_Other_ patients from the EMIF-AD cohort to the same analysis. **b** Multivariate analysis using PLS-DA (both cohorts) classified the two groups of MCI patients. **c** Variable importance in projection (VIP) scores for the top 15 most important proteins to discriminate MCI_AD_ from MCI_Other_. **d** GO enrichment analysis performed for “Decreased proteins” and “Increased proteins” showed enrichment for proteolysis, response to stimulus, complement activation, coagulation and response to wounding in both cohorts, among others. Similar or related Biological Processes have the same color. **e**, **f** PLS-DA classifying the different clusters of MCI_AD_ patients after a cluster analysis using nNMF performed for a two-cluster solution (**e**) and proteins that better discriminate the two clusters in each cohort (**f**). **g** GO analysis indicating two major subgroups of MCI_AD_ patients with decreased levels of proteins: one related to biological processes such as cell adhesion, coagulation, immune system and complement activation (Cluster 1) and the other to neurodevelopmental processes (Cluster 2) on both cohorts. **h** Comparison of AD biomarkers between Clusters from CCC (upper panel) and EMIF-AD (lower panel) cohorts. Box plots show that patients from Cluster 2 had higher levels of CSF pTau (left), CSF total Tau (t-tau, center) and CSF Aβ42 (right) as compared to Cluster 1 (independent sample *t*-test: *P < 0.05, **P < 0.01, ***P < 0.001)

CSF proteomics [6], generic functional analysis and gene ontology analysis (GO) of the quantified proteins showed similar biological pathways altered in patients from both cohorts (Additional file 1: Fig. S2 and Additional file 2: Tables S2-S4). By applying partial least squares discriminant analysis (PLS-DA), it was possible to accurately distinguish between MCI_AD_ and MCI_Other_ patient groups in the CCC cohort and, with less perfect discrimination, in the EMIF-AD cohort (Fig. 1b). This showed that the clinical criteria classification between the two groups of MCI patients can be translated into proteome alterations. Moreover, from PLS-DA we assessed the most important CSF set of proteins to distinguish MCI_AD_ from MCI_Other_. Proteins with high variable importance in projection (VIP) scores were regarded as significant and those with VIP > 1 were considered for further analysis. GO analysis performed on those proteins showed them to be related to biological processes already known to be altered in AD (Fig. 1d and Additional file 2: Table S5). Proteins that were shown to have decreased expression levels in MCI_AD_ patients compared to MCI_Other_ were mainly related to processes of coagulation, lipid metabolism, immune system, acute inflammatory response, and stress response; while proteins with increased levels in MCI_AD_ patients were related to energy and neurodevelopmental processes (Fig. 1c, d and Additional file 2: Table S6). Moreover, in the CCC cohort, NRP2 (neuropilin 2), APOA1 (apolipoporotein A I), AHSG/FETUA (alpha 2-HS glycoprotein), ORM1/A1AG1 (alpha-1-acid glycoprotein 1) and NBL1 (neuroblastoma suppressor of tumorigenicity 1) were identified as the most important CSF proteins to distinguish MCI_AD_ from MCI_Other_ patients, corresponding to the five highest VIP scores (Fig. 1c). These proteins, all being decreased in MCI_AD_ (Fig. 1c), have been previously described to be decreased in AD patients, including in the early phases of the disease, and are mainly related to the platelet degranulation pathway [7]. Platelets participate in several neuronal processes such as synaptic plasticity and contribute to the immune response. Platelet dysfunction has been pointed out as being implicated in several inflammatory and neurodegenerative disorders, including AD [8]. When analyzing the EMIF-AD cohort, five proteins emerged to best discriminate MCI_AD_ from MCI_Other_, GDIA (GDP dissociation inhibitor 1), ALDOA (aldolase, frutocse-biphosphate A), malate dehydrogenase (MDHC), ALDOC (aldolase, frutocse-biphosphate C) and GUAD (guanine deaminase). These proteins, which were increased in MCI_AD_ patients, have all been described as being associated with AD and are mainly related to glucose/pyruvate metabolism and neuronal function [7]. Several studies have shown that abnormal cerebral glucose metabolism is an early event before the pathological features of Aβ deposition [9, 10]. Though glucose metabolism is dramatically decreased in advanced AD, even being used as a biomarker and measured by the uptake of [^18^F]flurodeoxyglucose in PET, recent studies have shown an increase in glucose metabolism at early AD phases [9].

A nNMF (non-negative matrix factorization) clustering method was employed to explore the potential subtypes within MCI patients with AD pathology (Fig. 1e). The set of selected proteins from discriminant analysis was used to investigate possible proteomic differences that could indicate distinct underlying biological processes. We have investigated both possible approaches for two- and three-cluster solution. However, according to our fit criteria (Additional file 2: Table S7), for the resulting PLS-DA analysis and GO analysis of the different clusters of MCI_AD_ patients (Additional file 2: Tables S8–S11, and Additional file 1: Fig. S3 and S4), the two clusters could best describe the CSF proteomic data. Moreover, when performing an exploratory random forest classification of patients on the resulting two clusters, a small error was observed (< 9.5%) with both Cluster 1 and 2 being classified with high accuracy (> 85%) in the two cohorts. GO overrepresentation analysis was performed on the proteins with highest expression from Cluster 1 (83, 50.6%) and Cluster 2 (81, 49.4%) in the CCC cohort, and from Cluster 1 (101, 59.4%) and Cluster 2 (69, 40.6%) in the EMIF-AD cohort (Fig. 1g). For both cohorts, proteins from Cluster 1 were related to inflammatory and immune processes, including complement activation, together with haemostasis, coagulation, and fibrinolysis, and also regulation of peptidase, endopeptidase and hydrolase activities. Those same processes were related to the proteins with the lowest expression levels in Cluster 2. On the other hand, proteins with the highest expression level in Cluster 2 were related to neurodevelopmental processes, such as axonogenesis, neurogenesis and synapse organization, and to response to oxidative stress, which in turn were related to the proteins with the lowest level expression in Cluster 1. In the EMIF-AD cohort, proteins related to energy metabolism were also identified in Cluster 2. Remarkably, there was a statistically significant concordance between the two cohorts regarding the contribution of different gene ontologies to Cluster 1 and Cluster 2 (Cohen’s *kappa* = 0.398, *P* < 0.0001). All these findings suggest the existence of two subtypes of MCI_AD_ patients that could be described as blood-barrier dysfunction (Cluster 1) and hyperplasticity (Cluster 2), as previously proposed [3]. A comparison of the two MCI_AD_ clusters showed no differences in age, proportion of gender, education years and MMSE scores. However, CSF total Tau, CSF pTau and CSF Aβ_42_ levels were higher in Cluster 2 as compared to Cluster 1 (Fig. 1h, Additional file 1: Fig. S5 and Additional file 2: Table S12).

The biological processes associated with each of the two clusters of patients with MCI due to AD were quite similar between the two cohorts (Fig. 1g). However, the individual proteins selected in the discriminant analysis largely differed (Fig. 1f), suggesting alterations across various levels of common protein signaling cascades. Proteins in common between the two cohorts were analyzed to find a protein signature that could better identify the two MCI_AD_ subtypes in the analysis (Additional file 1: Fig. S6). Nine proteins exhibited the most distinct expression patterns, effectively distinguishing the two clusters with comparable expression profiles across both cohorts. AHSG/ FETUA, HEMO (hemopexin), ANT3, A2AP (alpha-2-antiplasmin), AFAM (afamin) and AMBP (alpha-1-microglobulin/bikinin precursor), associated with platelet degranulation and acute-inflammatory response, had higher expression levels in patients from Cluster 1, while PEBP1 (phpsphatidylethanolamine binding protein 1), MDHC and NCAN (neurocan), playing important roles in neurodevelopment and energy metabolism, showed higher expression levels in patients from Cluster 2. This potential protein signature might be worth further investigation since quantifying a limited set of proteins in the CSF may be enough to assign the patient to a specific cluster.

A limitation of the present study was the relatively small number of participants recruited from the CCC cohort. A strength was that patients with MCI fulfilling criteria of high likelihood for AD were compared to MCI patients without any biomarkers of Aβ deposition or neuronal injury, in order to control for nonspecific changes related to cognitive decline and lifestyle that might influence the proteome. The second strength of the study was the ability to replicate the results using an independent cohort from other countries.

Overall, our findings revealed the emergence of two main consistent subtypes of AD patients at the MCI stage, despite slight variations in diagnostic criteria, different sample preparation protocols, use of labelling (Tandem Mass Tag in the EMIF cohort) and being free of labelling (in the CCC cohort), as well as different LC–MS/MS quantification approaches (nano-DDA [data-dependent acquisition] in the EMIF and micro-DIA [data-independent acquisition] in the CCC cohorts). These subtypes can be described as (i) immune dysfunction and blood–brain barrier impairment (Cluster 1) and (ii) hyperplasticity (Cluster 2), as previously proposed. These findings may have significant implications for the design and interpretation of clinical trials, as there could be an association between treatment response and the specific AD subtype to which patients belong.

### Supplementary Information

**Additional file 1. **Supplementary materials and methods. **Figure S1.** General study overview. **Figure S2.** Biological pathways most represented by the analysed proteins in CCC and EMIF-AD cohorts are similar. **Figure S3.** Three cluster-solution analysis for CCC and EMIF-AD cohorts. **Figure S4.** Three cluster-solution analysis for the CCC and the EMIF-AD cohorts (cont.). **Figure S5.** AD biomarker comparison between Clusters from the CCC cohort. **Figure S6.** Clusters analysis of a common 55 proteins subset in both CCC and EMIF-AD cohorts.

**Additional file 2: Table S1.** Detailed participant description (with effect size). **Table S2.** Average (SD) protein levels for MCI groups of CCC (517 proteins) and EMIF-AD (570 proteins) cohorts and statistical analysis. **Table S3.** GO Reactome overrepresentation analysis for all 517 and 570 proteins from CCC and EMIF-AD cohorts, respectively, evaluated on this study. **Table S4.** GO Panther overrepresentation Fisher test for all proteins identified for CCC cohort (517) and evaluated in EMIF-AD cohort (570) with FDR correction. **Table S5.** Proteins resulting from the PLS-DA multivariate analysis for CCC (164) and EMIF-AD (170) cohorts. **Table S6.** GO Panther overrepresentation Fisher test for proteins resulting from PLS-DA analysis of CCC (164) and EMIF-AD (170) cohorts with FDR correction. **Table S7.** nNMF fit criteria on PLS-DA-selected proteins from each cohort. **Table S8.** Clusters from CCC and EMIF-AD cohorts. Three-cluster solution. **Table S9.** GO Panther overrepresentation Fisher test for proteins from each cluster from CCC or EMIF-AD cohort with FDR correction. Tree-cluster solution. **Table S10**. Clusters from CCC and EMIF-AD cohorts. Two-cluster solution. **Table S11.** GO Panther overrepresentation Fisher test for proteins from each cluster from CCC or EMIF-AD cohort with FDR correction. **Table S12.** Clinical and demographic comparisons of patients between clusters and cohorts.

## Data Availability

The mass spectrometry proteomics data have been deposited to the ProteomeXchange Consortium via the PRIDE partner repository with the dataset identifier PXD039563.

## Abbreviations

Aβ: Amyloid beta
AD: Alzheimer's disease
CCC: Cognitive Complaints Cohort
CSF: Cerebrospinal fluid
EMIF-AD: European Medical Information Framework for Alzheimer's Disease
GO: Gene ontology
MCI: Mild cognitive impairment
NRP2: Neuropilin 2
PLS-DA: Partial least squares discriminant analysis
VIP: Variable Importance in Projection
