# Supplementary material for: Pathophysiological subtypes of mild cognitive impairment due to Alzheimer’s disease identified by CSF proteomics
Source: Transl Neurodegener. 2024 Apr 9;13:19. doi: 10.1186/s40035-024-00412-1 (PMC11003166; doi:10.1186/s40035-024-00412-1)
Supplement: Supplementary file 1 — Additional file 1. Supplementary materials and methods. Figure S1. General study overview. Figure S2. Biological pathways most represented by the analysed proteins in CCC and EMIF-AD cohorts are similar. Figure S3. Three cluster-solution analysis for CCC and EMIF-AD cohorts. Figure S4. Three cluster-solution analysis for the CCC and the EMIF-AD cohorts (cont.). Figure S5. AD biomarker comparison between Clusters from the CCC cohort. Figure S6. Clusters analysis of a common 55 proteins subset in both CCC and EMIF-AD cohorts. [file 40035_2024_412_MOESM1_ESM.docx]

**Supplementary Materials and Methods**

*CCC Cohort description*

Participants were classified based on the criteria for mild cognitive impairment (MCI) due to AD adapted from the *National Institute on Aging - Alzheimer’s Association* workgroups (1). A total of 68 individuals with MCI were selected from CCC cohort to investigate proteomic changes at the early stages of Alzheimer’s disease (Figure S1 and Additional file 2: Table S1). Of those, 45 (66%) fulfilled high likelihood criteria for MCI due to AD (MCI_AD_), corresponding to the highest level of certainty for AD (1) fulfilling criteria 1, 2, 3 and 4 (Box 1). The other 23 (34%) subjects (MCI_Other_) fulfilled the clinical (criterion 1) and cognitive (criterion 2) criteria for MCI but showed none of the biomarkers of either Aβ deposition (criterion 3) or neuronal injury (criterion 4). Thus, participants with MCI_AD_ presented higher CSF p-tau and t-tau values, as well as lower CSF Aβ42 values than MCI_Other_ (Additional file 2: Table S1). Moreover, MCI_AD_ individuals were significantly older and had lower Mini Mental State Examination (MMSE) scores.

**Box 1. MCI due to AD criteria**

**(1) *Clinical and cognitive criteria***

a. Cognitive concern reflecting a change in cognition reported by patient, informant, or clinician

b. Objective evidence of impairment in one or more cognitive domains, typically including memory

c. Preservation of independence in functional abilities

d. Not demented

(**2) *Etiology of MCI consistent with AD pathophysiological process***

a. Vascular, traumatic and medical causes of cognitive decline were ruled out

b. Evidence of longitudinal decline in cognition (when feasible)

**(3) *Biomarkers of Aβ deposition***

a. Low CSF Aβ42

b. Positive amyloid PiB-PET imaging

**(4) *Biomarkers of neuronal injury***

a. High CSF total tau or hyperphosphorylated tau

b. Medial temporal atrophy by volumetric measures or visual rating

c. Temporoparietal hypometabolism by FDG-PET imaging

*CCC cohort CSF Proteomic analysis*

CSF proteomic analysis of the CCC cohort subjects was performed, resulting in the quantification of 517 proteins (Supplementary Table S2). Multivariate and gene ontology analysis approaches were then applied to analyze the proteomic profiles obtained. A generic functional analysis of these 517 quantified proteins was performed to identify which biological pathways are most represented by this set of proteins (Figure S2a, and Additional file 2: Tables S3 and S4).

To find a set of proteins that best discriminate between the two groups, the supervised multivariate Partial Least Square Discriminant Analysis (PLS-DA) was applied to min-max normalized, and scaled (mean-centered and divided by the standard deviation of each variable) protein expression data. A clear separation of the two MCI groups was achieved, except for 2 MCI_AD_ individuals (L05 and L56) that might be further considered as outliers (Figure 1b). From the initial 517 proteins in analysis, 164 proteins (31.7%; 150 downregulated and 14 upregulated) have shown to have a VIP score above one and were selected as the most important to distinguish between the two groups for further cluster analysis by nNMF (Additional file 2: Table S7).

The GO enrichment analysis of the 164 proteins resulting from PLS-DA shows that the 150 downregulated proteins are associated with several biological processes such as coagulation, fibrinolysis and haemostasis processes, as well as lipid metabolism, the immune system, including innate, humoral and adaptive immune responses, and inflammatory response, together with oxidative-stress processes, neuronal death, cell adhesion, extracellular matrix organization, signal transduction, and synapse, axon and neuron development (Figure 1d – “Decreased Proteins” and Additional file 2: Table S6). With an opposite trend, 14 proteins were found to be upregulated, associated with NADH, pyruvate, pyridine and carbohydrate metabolic processes and neuron differentiation (Figure 1d – “Increased Proteins”).

*EMIF-AD cohort description*

From the EMIF-AD cohort of 194 subjects with MCI, 92 (47.4%) were classified as MCI_AD_ and 102 (52.6%) as MCI_Other_, according to their CSF Aβ42 levels (Additional file 2: Table 1). As observed in the CCC cohort, MCI_AD_ individuals presented higher CSF p-tau and t-tau values, lower CSF Aβ42 levels, were significantly older and had lower MMSE scores than MCI_Other_.

*EMIF-AD cohort proteomic evaluation*

On the EMIF-AD cohort, a total of 2121 protein expression data was available for the 194 patients (Figure S1). Only the ones with no missing values for each subject were selected, resulting in an analysis of the expression data for 570 proteins. Generic functional analysis of these 570 identified proteins was performed to identify several pathways very similar to the pathways found for the CCC cohort (Figure S2b, and Additional file 2: Tables S3 and S4). To notice that among the initial 517 proteins from the CCC cohort and the 570 proteins from the EMIF-AD cohort, there were 391 proteins in common (Additional file 2: Table S2).

Protein expression data were log2-transformed, min-max-normalized, and mean-centered and divided by the standard deviation of each variable, before the multivariate PLS-DA was applied to the 194 EMIF-AD patients and their 570 proteins expression levels. A separation between the two groups of MCI individuals was observed, although not as clearly as for the CCC cohort (Figure 1b right panel). From the initial 570 proteins in analysis, 170 proteins (29.8%) have shown to have a VIP score above one (99 downregulated and 71 upregulated) and were selected as the most important to distinguish between the two groups for further cluster analysis by nNMF (Additional file 2: Table S5). Pathway enrichment analysis of the 170 proteins resulting from PLS-DA was performed on up-regulated proteins and down-regulated proteins, separately (Figure 1d and Additional file 2: Table S6).

In a preliminary phase, to increase the protein library used for protein quantification, 60 additional patients with MCI were selected according to similar clinical criteria from the CHUC cohort (Dementia Clinic, Neurology Department of Coimbra University Hospital, Coimbra, Portugal) and included in the pool and data extraction procedure.

*CCC Cohort: Cerebrospinal fluid proteomics*

SDS-PAGE: Protein precipitation was carried out by mixing 200 µL of CSF samples with 800 µL of methanol, at −80°C for about 1 hour followed by centrifugation at 20,000 × g for 20 minutes at 4°C. The protein pellet was resuspended in 50 µL of Laemmli sample buffer and sonicated for about 1min at 60% amplitude with a cup-horn (Ultrasonic processor, 750W) using cycles of 3 sec ON and 2 sec OFF. The total protein content was quantified by the Pierce™ 660nm Protein Assay Kit and different pools of samples for each condition (MCI_Other_ and MCI_AD_) containing 60 – 80 µg of protein were prepared and used to generate the peptide ion libraries (data-dependent acquisition - DDA). For the relative protein quantification (Data Independent Acquisition - SWATH/ DIA), 40 µg/sample of protein content were used for the sample processing. The protein content from each sample (60-80 µg DDA, 40 µg DIA) was separated by SDS-PAGE for about 17 minutes at 110 V (Short-GeLC Approach) and stained with Coomassie Brilliant Blue G-250 (3). For DDA experiments, each lane was divided into 5 gel pieces, and for DIA experiments into 3 gel pieces for further individual processing. After the distaining step, gel bands were incubated with trypsin for overnight protein digestion and peptides were extracted from the gel using 3 solutions containing different percentages of acetonitrile (30, 50, and 98%) with 1% formic acid. The organic solvent was evaporated using a vacuum concentrator and peptides were re-suspended in 25 µL (DDA) or 30 µL (DIA) with a solution containing 2% acetonitrile and 0.1% formic acid. Each sample was sonicated using a cup-horn (Ultrasonic processor, 750W) for about 2 minutes, 40% amplitude, and pulses of 1 sec ON/OFF. Ten microliters of each sample were analyzed by LC-MS/MS.

LC-MS data acquisition: Samples were analyzed on a NanoLC™ 425 System (Eksigent) coupled to a Triple TOF™ 6600 mass spectrometer (Sciex) equipped with an ESI DuoSpray™ Source (Sciex). The chromatographic separation was performed on a Triart C18 Capillary Column 1/32" (12 nm, S-3µm, 150 × 0.3 mm, YMC) and using a Triart C18 Capillary Guard Column (0.5 × 5 mm, 3 μm, 12nm, YMC) at 50°C. The flow rate was set to 5 µL/min and mobile phases A and B were 5% DMSO plus 0.1% formic acid in water and 5% DMSO plus 0.1% formic acid in acetonitrile, respectively. The LC program was performed as follows: 5 – 30% of B (0 - 50 min), 30 – 98% of B (50 – 52 min), 98% of B (52-54 min), 98 - 5% of B (54 – 56 min), and 5% of B (56 – 65 min). The ionization source was operated in the positive mode set to an ion spray voltage of 5500 V, 25 psi for nebulizer gas 1 (GS1), 10 psi for nebulizer gas 2 (GS2), 25 psi for the curtain gas (CUR), and source temperature (TEM) at 100°C. For DDA experiments, the mass spectrometer was set to scanning full spectra (m/z 350-2250) for 250 ms, followed by up to 100 MS/MS scans (m/z 100 – 1500). Candidate ions with a charge state between +1 and +5 and counts above a minimum threshold of 10 counts per second were isolated for fragmentation and one MS/MS spectrum was collected before adding those ions to the exclusion list for 15 seconds (mass spectrometer operated by Analyst® TF 1.8.1, Sciex®). The rolling collision was used with a collision energy spread of 5. For SWATH-DIA experiments, the mass spectrometer was operated in a looped product ion mode and specifically tuned to a set of 42 overlapping windows, covering the precursor mass range of 350-1400 m/z. A 50 ms survey scan (350-2250 m/z) was acquired at the beginning of each cycle, and SWATH-MS/MS spectra were collected from 100-2250 m/z for 50 ms resulting in a cycle time of 2.1 seconds.

Ion-Library (DDA information): A specific ion-library of the precursor masses and fragment ions was created by combining all files from the pools in one protein identification search using the ProteinPilot™ software (v5.0, Sciex). The paragon method parameters were the following: searched against the reviewed Human database from SwissProt (downloaded on May 15th of 2021), cysteine alkylation by acrylamide, digestion by trypsin, and gel-based ID. An independent False Discovery Rate (FDR) analysis, using the target-decoy approach provided by Protein Pilot™, was used to assess the quality of identifications.

Relative quantification of proteins (SWATH-MS): SWATH data processing was performed using SWATH™ processing plug-in for PeakView™ (v2.0.01, Sciex®). Protein relative quantification was performed in all samples using the information from the protein identification search. Quantification results were obtained for peptides with less than 1% of FDR and by the sum of up to 5 fragments/peptide. Each protein was normalized for the total sum of areas for the respective sample. Protein quantities were obtained by the sum of up to 15 peptides/protein.

The mass spectrometry proteomics data have been deposited to the ProteomeXchange Consortium via the PRIDE partner repository with the dataset identifier PXD039563.

*EMIF-AD Cohort: Cerebrospinal fluid proteomics*

Sample processing, data acquisition and data analysis are detailed in the work of Tijms et al, 2020 (4). The protein content of CSF samples was subjected to overnight liquid digestion by trypsin, followed by peptide labeling using the TMT approach. Subsequently, labeled peptides were fractionated through offline separation by High-pH Reversed-Phase chromatography, and fractions were analyzed by nano-LC coupled to a Q Exactive Orbitrap mass spectrometer. Data was acquired by data-dependent acquisition (DDA) for protein identification, with quantification relying on the reporter ion method.

Statistical Analysis

Statistical and classification analyses were performed using R 4.4 via RStudio and MetaboAnalyst 5.0 (<https://www.metaboanalyst.ca/>). MCI_AD_ and MCI_Other_ patients’ groups were compared for demographic characteristics and biomarker concentrations were performed with chi-square (for categorical variables), unpaired two-samples Student’s t-test (for continuous variables with normal distribution), and Mann Whitney U test (for continuous variables with non-normal distribution). Details on the statistical methods are described in the respective Table or Figure legend. Partial least square discriminant analysis (PLS-DA) for MCI_AD_ and MCI_Other_ groups comparison and random forest classification analysis for MCI_AD_ Cluster 1 and Cluster 2 patients’ comparison were performed in MetaboAnalyst 5.0. PLS-DA is a multivariate dimensionality reduction tool that takes into account class labels in order to identify which features have the greatest contribution for different sample group separation. It is a robust form of analysis, directed towards factor space that is associated with high variation in the responses but biased towards directions that are accurately predicted (in this case patient groups). Cluster analysis of MCI_AD_ patients was performed by non-negative matrix factorization (nNMF) to search for potential AD subtypes and is detailed in the next section. Random (decision) forest can be used to precisely classify observations and was used as a classification tool and a form of potentially evaluate clustering analysis resulting from nNMF. Concordance analysis on both cohorts GO analysis was performed applying Cohen’s kappa statistics. Statistical analyses for MCI_AD_ Cluster 1 and Cluster 2 comparison were performed on demographic characteristics and biomarker concentrations with chi-square (for categorical variables), unpaired two-samples Student’s t-tests (for continuous variables with normal distribution), and Mann Whitney U test (for continuous variables with non-normal distribution).

Cluster analysis with non-negative matrix factorization (nNMF)

Non-negative matrix factorization (nNMF) (5) is a clustering approach that organizes both the proteins and subjects to provide biological insight. This model is able to identify distinct protein patterns (protein profiles) and simultaneously groups subjects into subtypes based on the match of their protein expression levels to the protein patterns identified. Python 3.9 package Nimfa 1.4 (6) was used for clustering, with the function "Nsnmf" to perform the nonsmooth nonnegative matrix factorization and ensure sparse cluster solutions with higher separability. Since nNMF is an optimization algorithm that uses random initialization, the algorithm ran 50 times, and the model with the lowest objective function was retained. Two to five clusters were tested and selected the best number based on two criteria: (i) attaining a high value for the co-phonetic correlation; and (ii) a silhouette score of the cluster solution greater than 0.75.

System biology analysis

Panther Classification System 17.0 (<http://www.pantherdb.org>) online software was used for Gene Ontology (GO) statistical analysis to search for enriched biological processes. GO Term Finder (<https://go.princeton.edu/cgi-bin/GOTermFinderI>) online software was used to search for GO terms to apply on  REVIGO (<http://revigo.irb.hr/>) that was used to eliminate redundant GO terms. RStudio was used for graphical representation. Reactome online software (<https://reactome.org/>) was used to connect which pathways are associated with the identified proteins. UniProtKB (UniProt Protein Knowledgebase) and PubMed were consulted to obtain detailed information about relevant proteins and functions identified in the study.

**Supplementary Figures**

| 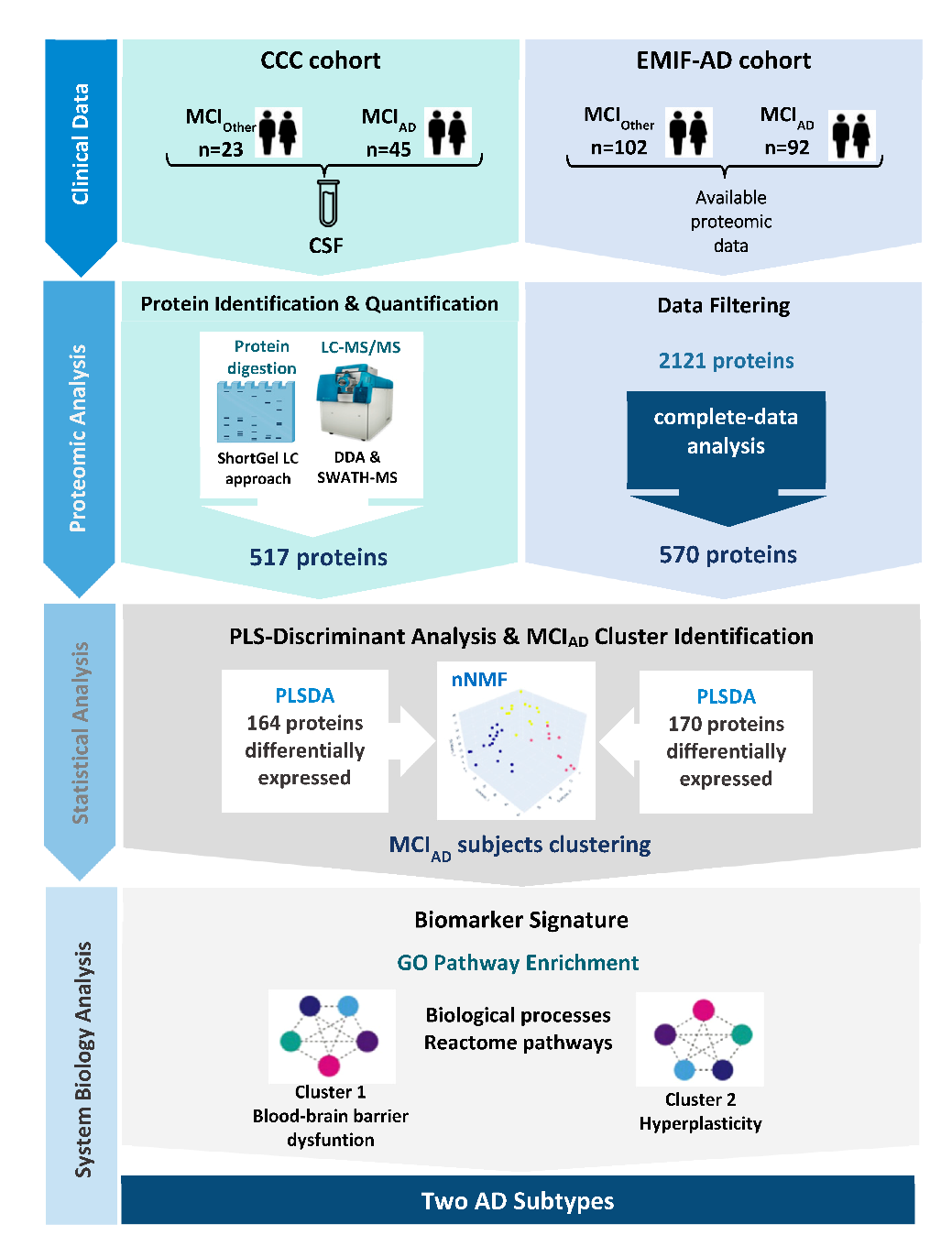 |
| --- |
| **Figure S1. General study overview**. CSF samples were obtained from a sample of 45 patients suffering from MCI due to AD (MCI_AD_) and 23 MCI patients showing no neurodegenerative biomarkers (MCI_Other_) from the CCC cohort. Proteomic characterization was done through short-Gel LC-SWATH-MS. Data analysis consisted of protein analysis approaches using partial least squares discriminant analysis (PLS-DA) followed by MCI_AD_ patients clustering analysis for subsequent subtype characterization through system biology analysis. Two clusters were identified: Cluster 1 showed increased levels of proteins related to biological processes as the immune system, acute inflammatory response, coagulation and lipid metabolism, whereas Cluster 2 showed involvement of neuronal and neurodevelopmental processes. Results were validated by submitting 92 MCI_AD_ and 102 MCI_Other_ patients’ proteomic data from EMIF-AD cohort to the same analysis. |
| 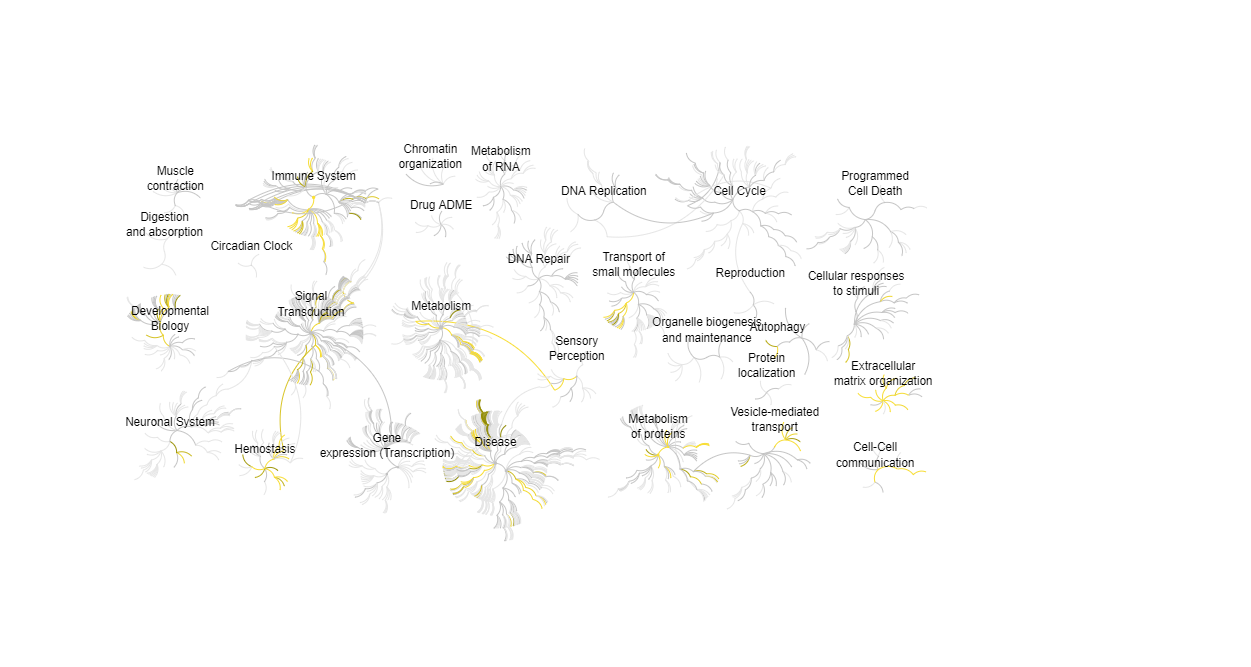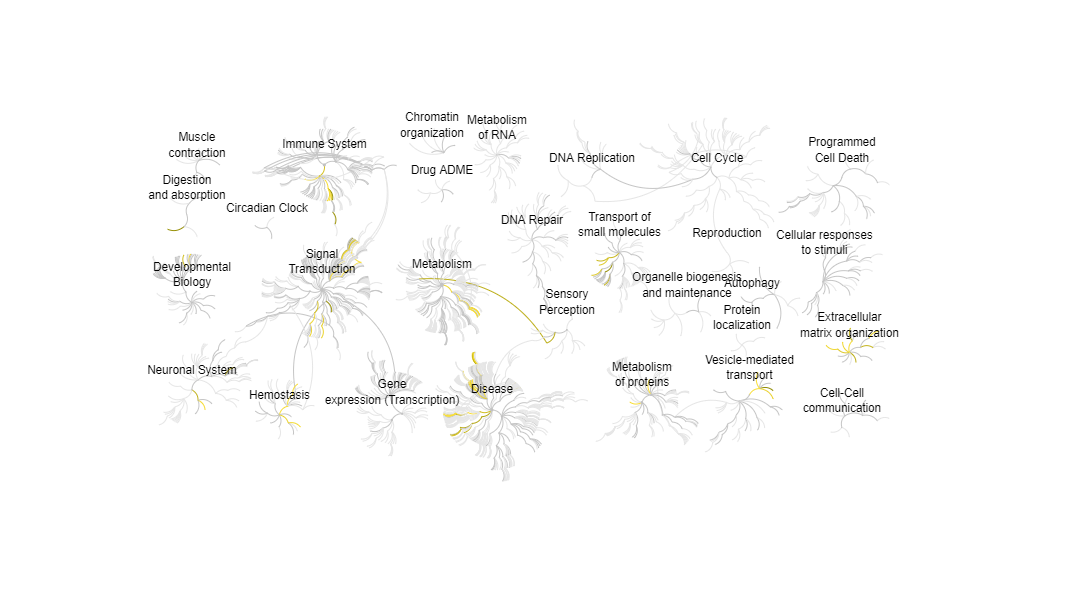 **a**  **b** |
| **Figure S2. Biological pathways most represented by the analysed proteins in CCC and EMIF-AD cohorts are similar. (a)** The 517 quantified proteins from the 68 MCI patients from CCC cohort showed to be most represented in several pathways involved in signal transduction, metabolism, developmental biology, neuronal system, extracellular matrix organization, immune system and haemostasis are over-represented by the identified proteins with significant enrichment, such as post-translational protein phosphorylation, regulation of insulin-like growth factor (IGF) transport, platelet degranulation, neutrophil degranulation, complement cascade, PI3K cascade or MAP2K and MAPK activation. **(b)** The 570 analysed proteins from the 194 MCI patients from EMIF-AD showed to be most represented in several pathways involved in signal transduction, developmental biology, neuronal system, metabolism, extracellular matrix organization, vesicle-mediated transport, immune system and haemostasis as being over-represented by the identified proteins with significant enrichment, very similar to the pathways found for the CCC cohort (Additional file 2: Table S3) |


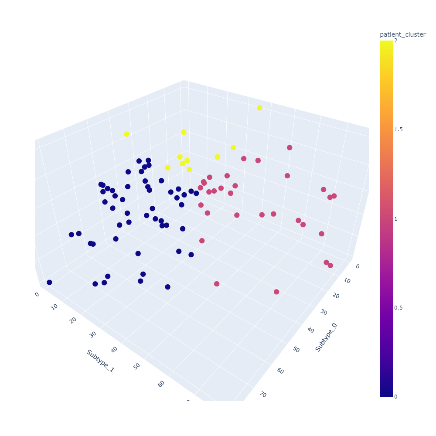

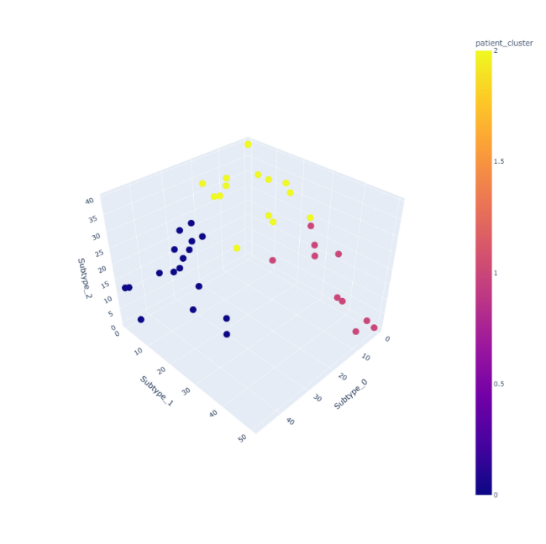


**a CCC b EMIF-AD g CCC h EMIF-AD**


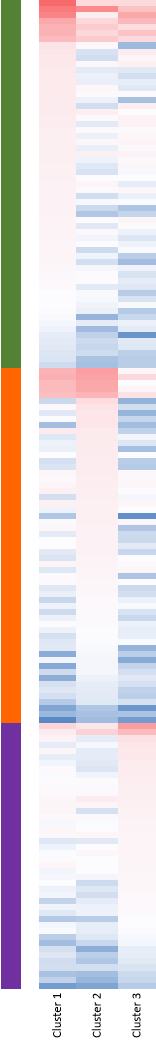

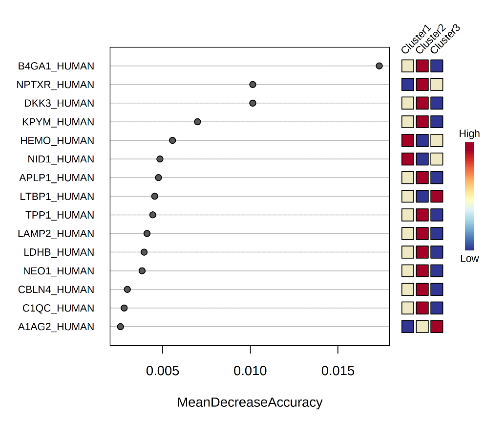

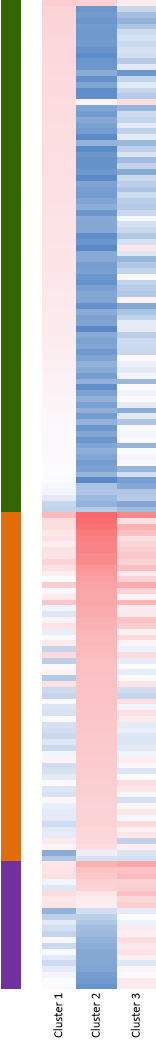

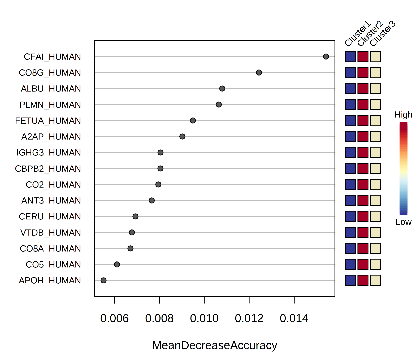


**c CCC d EMIF-AD**

**e CCC f EMIF-AD**


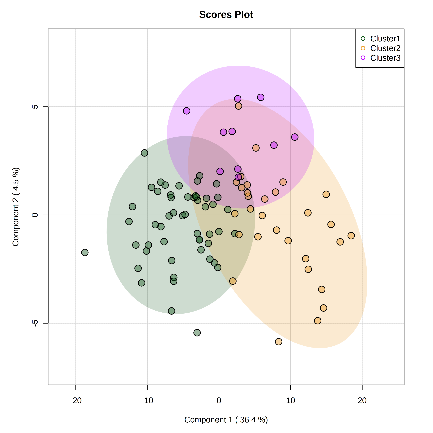

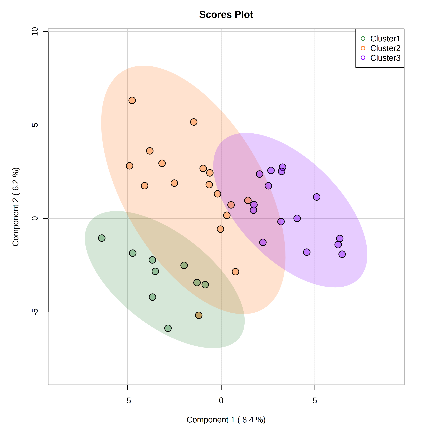


**Figure S3. Three cluster-solution analysis for CCC and EMIF-AD cohorts**. An analysis was performed for a solution of three clusters with the representation of (a and b) the subject loading on subtype scores for both cohorts, indicating which cluster each individual (dot) fits best. A PLS-DA (c and d) was used to classify the different clusters of MCI_AD_ patients and an exploratory random forest classification (e and f) showed which proteins better discriminate between clusters in each cluster solution. A heatmap (g and h) of the cluster average Z-scores (relative to MCI_Other_) shows how proteins organize in each cluster (see Supplementary Table S9 for the list of proteins).

**e**


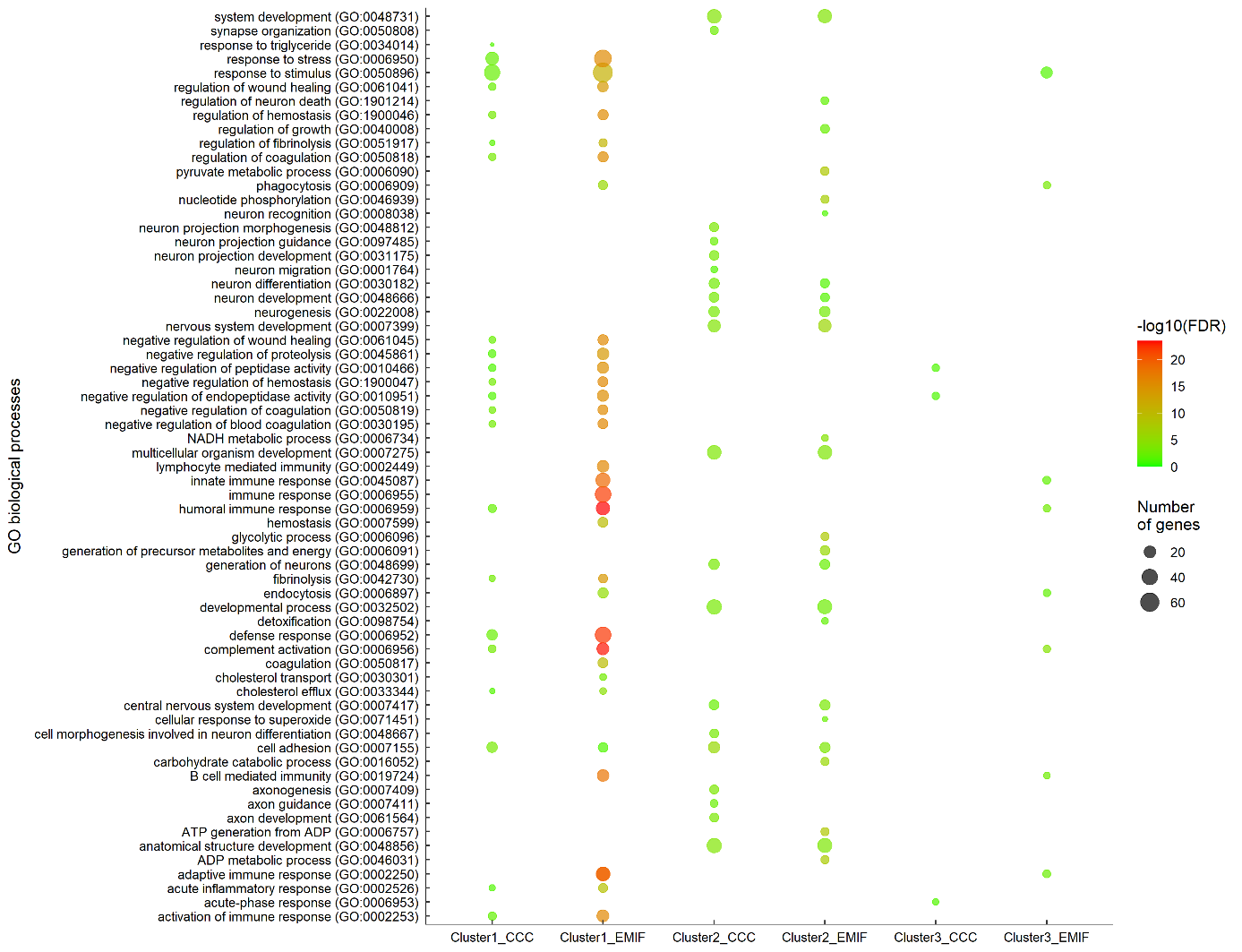


**CCC EMIF-AD CCC EMIF-AD CCC EMIF-AD**

**Cluster 1 Cluster 2 Cluster 3**

|  |
| --- |
| **Figure S4. Three cluster-solution analysis for the CCC and EMIF-AD cohorts (cont.)**. A gene ontology analysis shows enrichment for multiple ontologies, indicating two major subgroups of patients with decreased levels of proteins: one related to biological processes as cell adhesion, coagulation, immune system and complement activation (Cluster 1) and the other to neurodevelopmental processes (Cluster 2). The Cluster 3, for both cohorts, showed to be related to the same pathways observed for Cluster 1, suggesting that a two-cluster solution should fit best for both cohorts. See Supplementary Table S10 for the full GO biological processes list. |

| **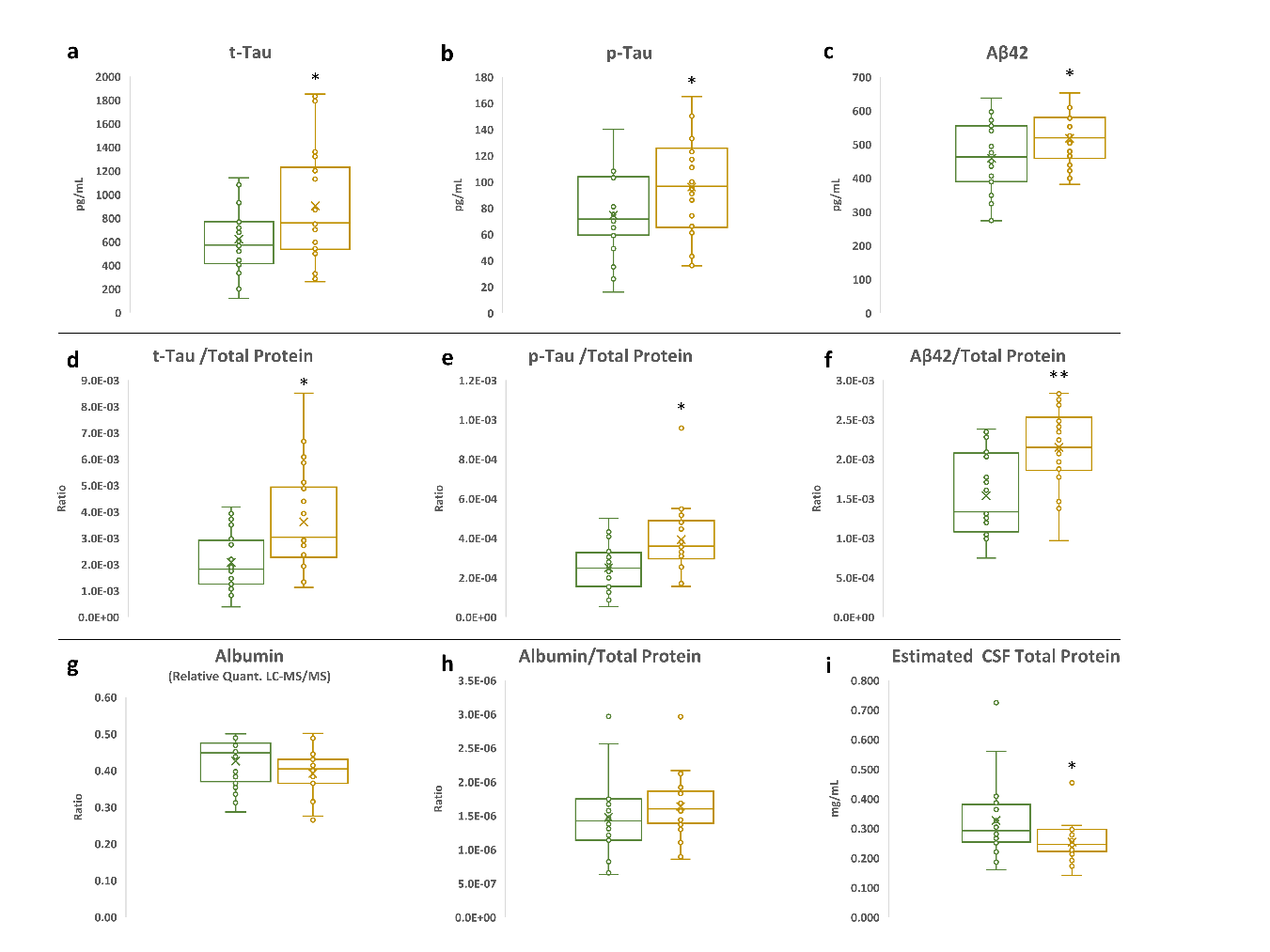** |
| --- |
| **Figure S5. AD biomarkers comparison between Clusters from CCC cohort.** Two independent sample t-test was applied to patients from each cluster (a) CSF tTau, (b) pTau, and (c) Aβ42 levels. The ratio between (d) CSF tTau and CSF total protein, (e) CSF pTau and CSF total protein, and (f) CSF Aβ42 and CSF total proteins was also investigated. The levels of (c) CSF albumin, (d) ratio of CSF albumin and CSF total protein ratio, and (e) CSF total protein were also estimated. Significant differences in levels are indicated (* p<0.05, ** *p* <0.01). |

| \|  \|  \| **CCC** \| \| **EMIF** \| \|  \| \| --- \| --- \| --- \| --- \| --- \| --- \| --- \| \|  \|  \|  \|  \|  \|  \| NID2_HUMAN \| \|  \|  \|  \|  \|  \|  \| CNTP2_HUMAN \| \|  \|  \|  \|  \|  \|  \| KLKB1_HUMAN \| \|  \|  \|  \|  \|  \|  \| ALBU_HUMAN \| \|  \|  \|  \|  \|  \|  \| KNG1_HUMAN \| \|  \|  \|  \|  \|  \|  \| A2AP_HUMAN \| \|  \|  \|  \|  \|  \|  \| FIBG_HUMAN \| \|  \|  \|  \|  \|  \|  \| APOB_HUMAN \| \|  \|  \|  \|  \|  \|  \| AFAM_HUMAN \| \|  \|  \|  \|  \|  \|  \| LV39_HUMAN \| \|  \|  \|  \|  \|  \|  \| C4BPA_HUMAN \| \|  \|  \|  \|  \|  \|  \| CFAB_HUMAN \| \|  \|  \|  \|  \|  \|  \| HEMO_HUMAN \| \|  \|  \|  \|  \|  \|  \| H6ST3_HUMAN \| \|  \|  \|  \|  \|  \|  \| IGA2_HUMAN \| \|  \|  \|  \|  \|  \|  \| THBG_HUMAN \| \|  \|  \|  \|  \|  \|  \| IGK_HUMAN \| \|  \|  \|  \|  \|  \|  \| LV310_HUMAN \| \|  \|  \|  \|  \|  \|  \| CBG_HUMAN \| \|  \|  \|  \|  \|  \|  \| FGFR2_HUMAN \| \|  \|  \|  \|  \|  \|  \| CFAI_HUMAN \| \|  \|  \|  \|  \|  \|  \| AMBP_HUMAN \| \|  \|  \|  \|  \|  \|  \| SPON1_HUMAN \| \|  \|  \|  \|  \|  \|  \| A1AG2_HUMAN \| \|  \|  \|  \|  \|  \|  \| GDIA_HUMAN \| \|  \|  \|  \|  \|  \|  \| ITIH1_HUMAN \| \|  \|  \|  \|  \|  \|  \| LTBP4_HUMAN \| \|  \|  \|  \|  \|  \|  \| ANT3_HUMAN \| \|  \|  \|  \|  \|  \|  \| ALS_HUMAN \| \|  \|  \|  \|  \|  \|  \| CERU_HUMAN \| \|  \|  \|  \|  \|  \|  \| HRG_HUMAN \| \|  \|  \|  \|  \|  \|  \| APOL1_HUMAN \| \|  \|  \|  \|  \|  \|  \| ITIH4_HUMAN \| \|  \|  \|  \|  \|  \|  \| A1AG1_HUMAN \| \|  \|  \|  \|  \|  \|  \| APOA1_HUMAN \| \|  \|  \|  \|  \|  \|  \| FETUA_HUMAN \| \|  \|  \|  \|  \|  \|  \| LDHB_HUMAN \| \|  \|  \|  \|  \|  \|  \| CH3L1_HUMAN \| \|  \|  \|  \|  \|  \|  \| KPYM_HUMAN \| \|  \|  \|  \|  \|  \|  \| MDHC_HUMAN \| \|  \|  \|  \|  \|  \|  \| TRFE_HUMAN \| \|  \|  \|  \|  \|  \|  \| PTPRZ_HUMAN \| \|  \|  \|  \|  \|  \|  \| NCAN_HUMAN \| \|  \|  \|  \|  \|  \|  \| TAGL_HUMAN \| \|  \|  \|  \|  \|  \|  \| FGFR1_HUMAN \| \|  \|  \|  \|  \|  \|  \| SODM_HUMAN \| \|  \|  \|  \|  \|  \|  \| NRX1A_HUMAN \| \|  \|  \|  \|  \|  \|  \| PEBP1_HUMAN \| \|  \|  \|  \|  \|  \|  \| CHRD_HUMAN \| \|  \|  \|  \|  \|  \|  \| SAA4_HUMAN \| \|  \|  \|  \|  \|  \|  \| KCC2A_HUMAN \| \|  \|  \|  \|  \|  \|  \| CBPB2_HUMAN \| \|  \|  \|  \|  \|  \|  \| C1RL_HUMAN \| \|  \|  \|  \|  \|  \|  \| ZA2G_HUMAN \| \|  \|  \|  \|  \|  \|  \| APOA4_HUMAN \| \|  \|  \| **Cluster 1** \| **Cluster 2** \| **Cluster 1** \| **Cluster 2** \|  \|  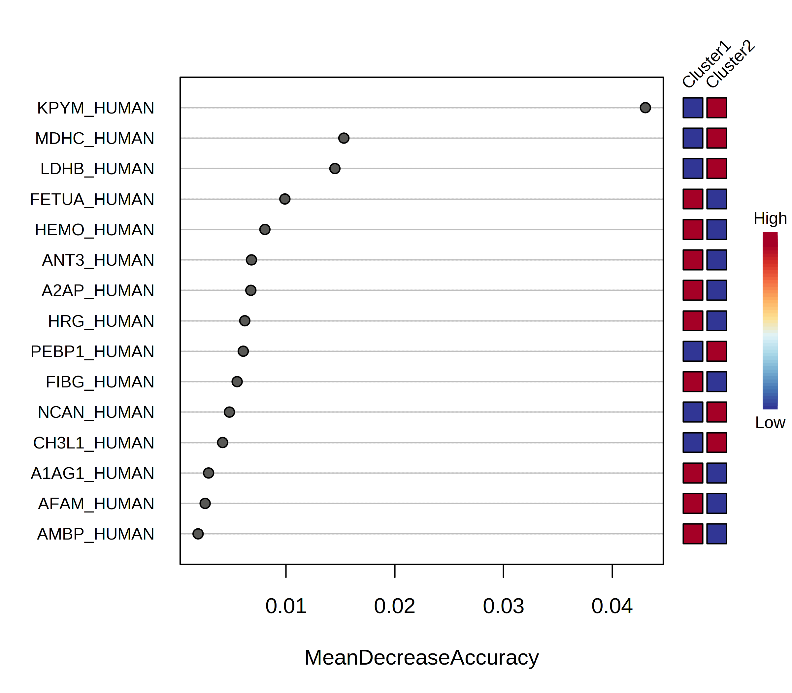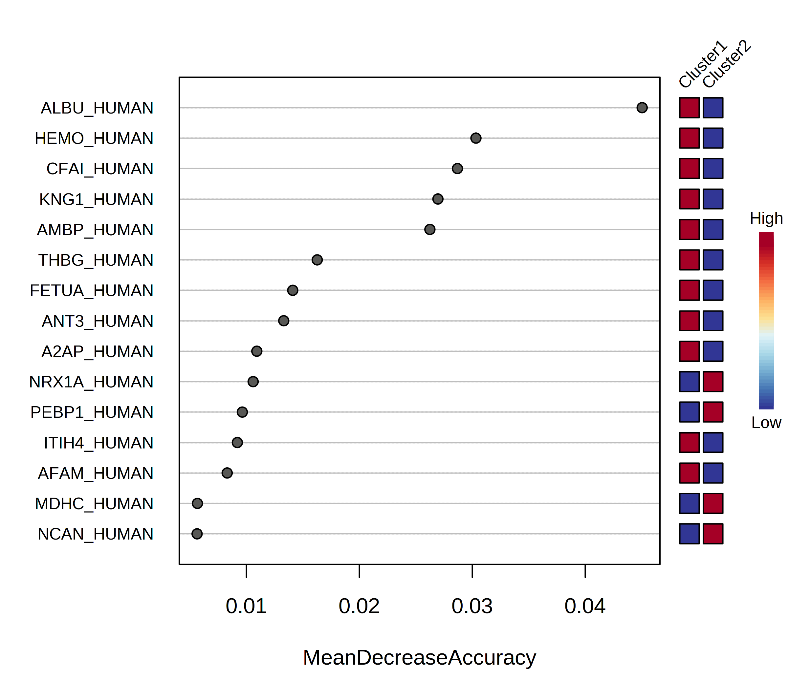 **CCC**  **EMIF-AD**  **a**  **b**  **c** |
| --- | --- | --- | --- | --- | --- | --- | --- | --- | --- | --- | --- | --- | --- | --- | --- | --- | --- | --- | --- | --- | --- | --- | --- | --- | --- | --- | --- | --- | --- | --- | --- | --- | --- | --- | --- | --- | --- | --- | --- | --- | --- | --- | --- | --- | --- | --- | --- | --- | --- | --- | --- | --- | --- | --- | --- | --- | --- | --- | --- | --- | --- | --- | --- | --- | --- | --- | --- | --- | --- | --- | --- | --- | --- | --- | --- | --- | --- | --- | --- | --- | --- | --- | --- | --- | --- | --- | --- | --- | --- | --- | --- | --- | --- | --- | --- | --- | --- | --- | --- | --- | --- | --- | --- | --- | --- | --- | --- | --- | --- | --- | --- | --- | --- | --- | --- | --- | --- | --- | --- | --- | --- | --- | --- | --- | --- | --- | --- | --- | --- | --- | --- | --- | --- | --- | --- | --- | --- | --- | --- | --- | --- | --- | --- | --- | --- | --- | --- | --- | --- | --- | --- | --- | --- | --- | --- | --- | --- | --- | --- | --- | --- | --- | --- | --- | --- | --- | --- | --- | --- | --- | --- | --- | --- | --- | --- | --- | --- | --- | --- | --- | --- | --- | --- | --- | --- | --- | --- | --- | --- | --- | --- | --- | --- | --- | --- | --- | --- | --- | --- | --- | --- | --- | --- | --- | --- | --- | --- | --- | --- | --- | --- | --- | --- | --- | --- | --- | --- | --- | --- | --- | --- | --- | --- | --- | --- | --- | --- | --- | --- | --- | --- | --- | --- | --- | --- | --- | --- | --- | --- | --- | --- | --- | --- | --- | --- | --- | --- | --- | --- | --- | --- | --- | --- | --- | --- | --- | --- | --- | --- | --- | --- | --- | --- | --- | --- | --- | --- | --- | --- | --- | --- | --- | --- | --- | --- | --- | --- | --- | --- | --- | --- | --- | --- | --- | --- | --- | --- | --- | --- | --- | --- | --- | --- | --- | --- | --- | --- | --- | --- | --- | --- | --- | --- | --- | --- | --- | --- | --- | --- | --- | --- | --- | --- | --- | --- | --- | --- | --- | --- | --- | --- | --- | --- | --- | --- | --- | --- | --- | --- | --- | --- | --- | --- | --- | --- | --- | --- | --- | --- | --- | --- | --- | --- | --- | --- | --- | --- | --- | --- | --- | --- | --- | --- | --- | --- | --- | --- | --- | --- | --- | --- | --- | --- | --- | --- | --- | --- | --- | --- | --- | --- | --- | --- | --- | --- | --- | --- | --- | --- | --- | --- | --- | --- | --- | --- | --- | --- | --- | --- | --- | --- | --- | --- | --- | --- | --- | --- | --- | --- |
| **Figure S6. Clusters analysis of a common 55 proteins subset in both CCC and EMIF-AD cohorts.**  Proteins average levels were compared between clusters and cohorts by (**a**) the Z-scores according to the mean and standard deviation of the controls within each cohort represented in a heatmap. An exploratory random forest was performed on this protein subset to check which proteins best separate the two clusters in (**b**) CCC and (**c**) EMIF-AD cohorts. HEMO, FETUA, ANT3, A2AP, PEBP1, followed by AMBP, MDHC, AFAM and NCAN were the most consistent proteins across both cohorts’ analysis. |

**References**

1. Albert MS, DeKosky ST, Dickson D, Dubois B, Feldman HH, Fox NC, et al. The diagnosis of mild cognitive impairment due to Alzheimer’s disease: recommendations from the National Institute on Aging-Alzheimer’s Association workgroups on diagnostic guidelines for Alzheimer’s disease. Alzheimers Dement [Internet]. 2011 [cited 2022 Aug 26];7(3):270–9. Available from: https://pubmed.ncbi.nlm.nih.gov/21514249/

2. Baldeiras I, Santana I, Proença MT, Garrucho MH, Pascoal R, Rodrigues A, et al. Oxidative damage and progression to Alzheimer’s disease in patients with mild cognitive impairment. J Alzheimer’s Dis [Internet]. 2010 [cited 2023 Feb 16];21(4):1165–77. Available from: https://pubmed.ncbi.nlm.nih.gov/21504121/

3. Manadas B, Santos AR, Szabadfi K, Gomes JR, Garbis SD, Fountoulakis M, et al. BDNF-induced changes in the expression of the translation machinery in hippocampal neurons: Protein levels and dendritic mRNA. J Proteome Res [Internet]. 2009 [cited 2023 Jan 9];8(10):4536–52. Available from: https://pubmed.ncbi.nlm.nih.gov/19702335/

4. Tijms BM, Gobom J, Reus L, Jansen I, Hong S, Dobricic V, et al. Pathophysiological subtypes of Alzheimer’s disease based on cerebrospinal fluid proteomics. Brain [Internet]. 2020 Dec 1 [cited 2022 Jul 5];143(12):3776–92. Available from: https://pubmed.ncbi.nlm.nih.gov/33439986/

5. Lee DD, Seung HS. Learning the parts of objects by non-negative matrix factorization. Nat 1999 4016755 [Internet]. 1999 Oct 21 [cited 2023 Jan 6];401(6755):788–91. Available from: https://www.nature.com/articles/44565

6. Marinkažitnik MM, Zupan B. NIMFA : A Python Library for Nonnegative Matrix Factorization. J Mach Learn Res [Internet]. 2012 [cited 2023 Jan 6];13:849–53. Available from: http://code.google.com/p/
